# Supplementary material for: Splice-Junction-Based Mapping of Alternative Isoforms in the Human Proteome
Source: Cell Rep. Author manuscript; Available in PMC 2020 Jan 15. (PMC6961840; doi:10.1016/j.celrep.2019.11.026)
Supplement: 2 [file NIHMS1546469-supplement-2.zip › DF1/PXD010154/Heart/Heart_22_TTN_RVEAEPAEEVTIMEEK.pdf]

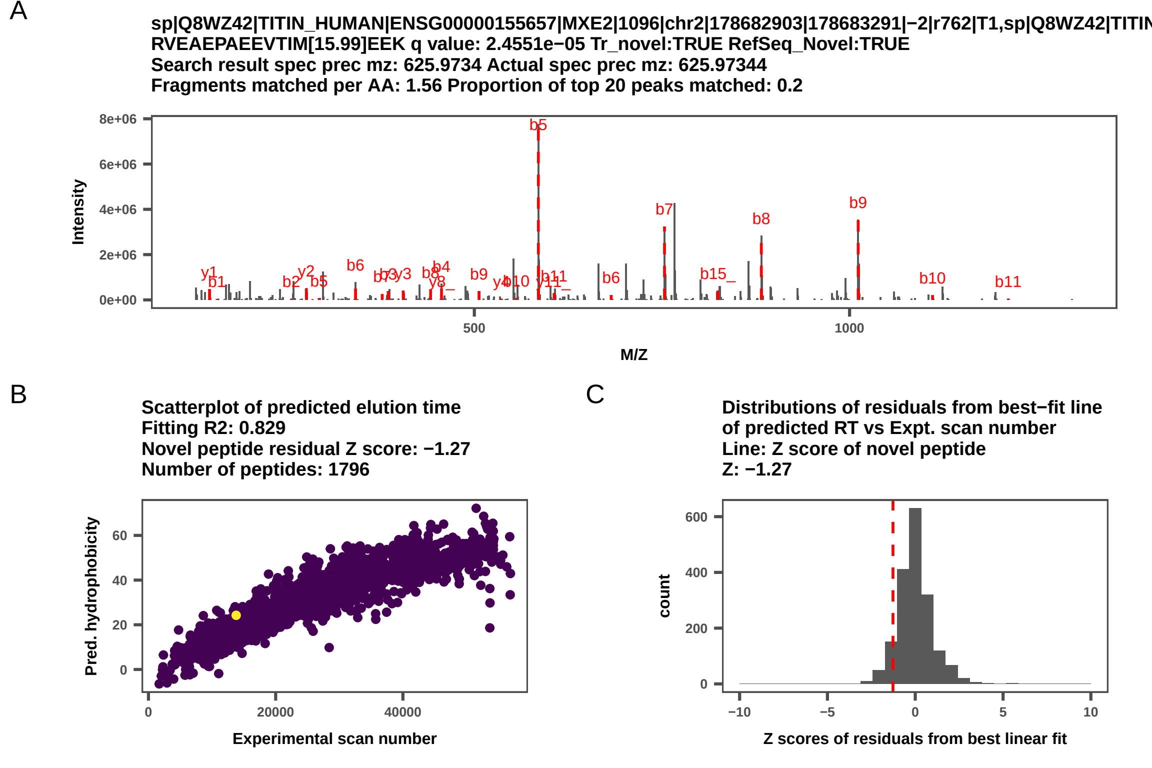

A
sp|Q8WZ42|TITIN_HUMAN|ENSG00000155657|MXE2|1096|chr2|178682903|178683291|−2|r762|T1,sp|Q8WZ42|TITIN
RVEAEPAEEVTIM[15.99]EEK q value: 2.4551e−05 Tr_novel:TRUE RefSeq_Novel:TRUE
Search result spec prec mz: 625.9734 Actual spec prec mz: 625.97344
Fragments matched per AA: 1.56 Proportion of top 20 peaks matched: 0.2
Intensity
M/Z
B
Scatterplot of predicted elution time
Fitting R2: 0.829
Novel peptide residual Z score: −1.27
Number of peptides: 1796
Pred. hydrophobicity
Experimental scan number
C
Distributions of residuals from best−fit line
of predicted RT vs Expt. scan number
Line: Z score of novel peptide
Z: −1.27
count
Z scores of residuals from best linear fit
